# Coordinated Regulation of PPAR*γ* Expression and Activity through Control of Chromatin Structure in Adipogenesis and Obesity

**DOI:** 10.1155/2012/164140

**Published:** 2012-09-06

**Authors:** Jérôme Eeckhoute, Frédérik Oger, Bart Staels, Philippe Lefebvre

**Affiliations:** ^1^Université Lille Nord de France, 59000 Lille, France; ^2^Inserm, U1011, 59000 Lille, France; ^3^UDSL, 59000 Lille, France; ^4^Institut Pasteur de Lille, 59019 Lille, France

## Abstract

The nuclear receptor peroxisome proliferator-activated receptor gamma (PPAR**γ**) is required for differentiation and function of mature adipocytes. Its expression is induced during adipogenesis where it plays a key role in establishing the transcriptome of terminally differentiated white fat cells. Here, we review findings indicating that PPAR**γ** expression and activity are intricately regulated through control of chromatin structure. Hierarchical and combinatorial activation of transcription factors, noncoding RNAs, and chromatin remodelers allows for temporally controlled expression of PPAR**γ** and its target genes through sequential chromatin remodelling. In obesity, these regulatory pathways may be altered and lead to modified PPAR**γ** activity.

## 1. Introduction

Peroxisome proliferator-activated receptor gamma (PPAR*γ*) is a transcription factor (TF) belonging to the superfamily of nuclear receptors. PPAR*γ* has a well-established central role in differentiation and function of mature adipocytes [1–5]. This nuclear receptor is activated by endogenous ligands such as nitrated linoleic acid or oxidized fatty acids (9- and 13-HODE), prostaglandin J2 as well as by various synthetic ligands, including the insulin sensitizers pioglitazone and rosiglitazone [6]. Indeed, activation of PPAR*γ* by the thiazolidinediones (TZDs) pioglitazone or rosiglitazone improves insulin resistance associated with obesity and diabetes [7]. Recent studies suggest that this effect is mainly mediated through activation of PPAR*γ* in adipocytes [4, 8], although studies also suggest that macrophage [9] and brain PPAR*γ* [10] contributes to this therapeutic effect.

Transcriptional regulation in mammalian cells is intimately linked to the genomic organisation of the DNA in a highly dynamic chromatin structure. Indeed, chromatin intrinsically impedes transcription factor access to the DNA. This is illustrated by the finding that TFs bind to a highly limited number of potential response elements within the genome of eukaryotic cells [11]. Typically, chromatin regions bound by TFs are devoid of nucleosomes since they can mask their DNA recognition motifs [12]. An additional layer of regulation is provided by epigenomic signatures including histone variant incorporation, histone posttranslational modifications as well as DNA (hydroxy)methylation. Transcriptional regulators and epigenetic modifications mutually regulate each other in order to achieve proper cell type and environment-specific usage of all functional DNA sites embedded within the genome [13–15]. Hence, transcriptional regulatory regions are characterized by different epigenetic signatures when inactive, poised, or active [16, 17]. 

In this context, results from several biochemical and genetic studies led to the concept that many transcription factors, including nuclear receptors, require presetting of the chromatin for binding to their response elements in the DNA sequence [13, 15]. This consists of preliminary remodelling of the chromatin landscape allowing subsequent TF binding. This process involves so-called pioneer factors that can be recruited to nucleosomal DNA. Such pioneer factors would be required to initiate chromatin remodelling and competency of enhancers that are subsequently used by TFs to mediate transcriptional regulatory signals. Strikingly, some TFs bearing these functions can remain associated with mitotic chromatin suggesting they could bookmark regulatory sites and represent “epigenetic cues” [13, 15].

During adipocyte differentiation, chromatin remodelling events take place to allow proper PPAR*γ* expression and activity. We review here our current knowledge of an integrated control of PPAR*γ* expression and function in adipocytes emphasizing the central role of chromatin remodelling. 

## 2. Regulation of *PPAR*γ** Gene Expression in Adipocytes

### 2.1. Transcriptional Regulation of PPAR*γ*

The study of adipogenesis and adipocyte functions has benefited from cellular models which can be easily manipulated *in vitro* [18, 19]. Numerous studies made use of the mouse preadipocyte 3T3-L1 cell-line, which can adopt an adipocyte-like phenotype with lipid droplet accumulation upon stimulation with a cocktail of adipogenic inducers (isobutylmethylxanthine (IBMX), dexamethasone, and insulin). During this process, PPAR*γ* expression is strongly induced. Two isoforms of PPAR*γ* are encoded from alternative promoters in the mouse, namely, PPAR*γ*1 and PPAR*γ*2. While PPAR*γ*1 is found in numerous tissues, PPAR*γ*2 expression is mostly restricted to white and brown adipose tissues [20]. PPAR*γ*2 possesses 30 additional aminoacids, which renders the PPAR*γ* aminoterminal transactivation domain more active [21, 22]. Thus, while both PPAR*γ* isoforms can induce adipogenesis, PPAR*γ*2 is thought to play a dominant role in this process [23, 24]. 

Very early during adipogenesis, the expression of ecotropic viral integration site 1 (Evi1), CCAAT/enhancer binding protein (C/EBP) *β* and C/EBP*δ* is induced [25, 26]. This results in expression of low levels of the two PPAR*γ* isoforms and of C/EBP*α* [25, 26] maintained in a repressed state in preadipocytes by the transcriptional corepressor SMRT (silencing mediator of retinoic acid and thyroid hormone receptor) [27]. PPAR*γ* and C/EBP*α* can then induce each other's expression in a positive feedback loop promoting and maintaining the differentiated state of the adipocyte [3]. Interestingly, genomic profiling of PPAR*γ* binding sites in adipocytes has revealed that it is present both at the PPAR*γ*2 promoter and at potential enhancers in the vicinity or within its own gene [28, 29]. Expression of PPAR*γ* also requires the activity of the krüppel-like factors 5 and 15 (KLF5 and KLF15) secondarily to their induction by C/EBP transcription factors [30, 31]. Additionally, the transcription factors nuclear family I (NFI) and nuclear factor E2-related factor 2 (Nrf2) regulate both C/EBP*α* and PPAR*γ* during adipogenesis most probably through direct binding to the PPAR*γ*1 and PPAR*γ*2 promoter, respectively [32, 33]. Additionally, NFI could exert its activities through binding to enhancers within or near both genes [33]. 

Gene expression is induced by TFs and their cofactors through chromatin remodelling events triggered by cofactor enzymatic activities catalyzing histone and DNA modifications [13–15]. Indeed, the transcriptional activation of PPAR*γ* during adipogenesis correlates with an epigenetic switch at the *PPAR*γ** gene. For instance, adipocyte differentiation is associated with a strong increase in levels of histone activation marks at the two PPAR*γ* promoters. This includes acetylation of histone H3 lysine 27 (H3K27ac) and methylation of H3K4 (H3K4me2/3) and H4K20 (H4K20me1) [29, 34]. H3K27ac, which is catalyzed by the transcriptional coactivators CREB-binding protein (CBP) and p300 [35] and typically found at active transcriptional regulatory regions, also increases at enhancers within or near the *PPAR*γ** gene [28]. Activation of the PPAR*γ* promoters is also associated with the removal of repressive marks including H3K9me2 and H3K27me3 [17, 34, 36, 37]. The switch from methylation to acetylation at H3K27 could therefore represent a point of integration between activating and repressing signals. Concomitantly, demethylation of the PPAR*γ*2 promoter, which leads to the release of the transcriptional inhibitor methyl CpG-binding protein 2 (MeCP2), occurs gradually during differentiation paralleling the continuing rise in PPAR*γ*2 mRNA expression [34, 38]. 

**Figure 1:**
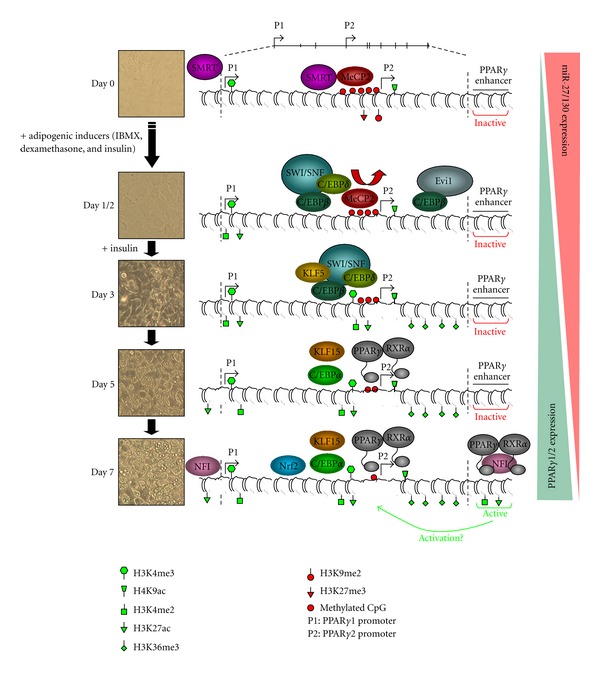
Chromatin-based regulation of PPAR*γ* expression in adipogenesis. Schematic describing epigenomic events as well as sequential transcription factor binding involved in the regulation of *PPAR*γ*1/2* expression during adipogenesis. Expression of miR-27/130 targeting *PPAR*γ** is also indicated.

These epigenetic changes create an environment competent for gene induction. However, additional remodelling is required. Indeed, PPAR*γ* promoters also undergo chromatin reconfiguration through the binding of the nucleosome-remodeling complex switch/sucrose non-fermentable (SWI/SNF) [39]. SWI/SNF is required neither for epigenetic changes nor for general TF recruitment, but promotes transcription elongation [39]. Hence, PPAR*γ* induction is a multistep process in which sequential chromatin remodelling events eventually lead to the release of stalled RNA polymerase II. Control of transcription elongation through modulation of RNA polymerase II release from promoters has recently emerged as a central mechanism governing developmental gene expression [40]. Like developmental gene promoters in pluripotent cells [41], the PPAR*γ*1 promoter bears H3K4me3 in preadipocytes [28], which could facilitate its induction during differentiation [41]. RNA polymerase II stalling is not a mere consequence of transcription regulation but is by itself an integral part of gene regulation by competing with nucleosomes at promoters and therefore setting the ground for induction [40, 42]. Finally, release of stalled RNA polymerase II results in trimethylation of H3K36 within the *PPAR*γ** gene, a feature of actively transcribed regions (Figure 1) [28, 43].

The function of adipose tissues is severely altered in obesity [44]. However, this does not stem from a reduced expression of PPAR*γ*, which remains unchanged or increased in adipose tissues from obese rats, mice, and humans [45–48]. Accordingly, knock-out of Nrf2 decreases PPAR*γ* expression, impairs adipogenesis, and protects mice from obesity [32]. Sustained expression of PPAR*γ* in WAT of obese mice may involve a decrease in levels of the orphan nuclear receptor chicken ovalbumin upstream promoter transcription factor II (COUP-TFII), which represses transcription of the *PPAR*γ** gene by bringing SMRT and decreasing histone acetylation levels at its promoters [49]. Note however that COUP-TFII role in adipogenesis is still to be clarified since contradictory results have been reported [50, 51]. In this context, perturbation of adipocyte functions in obesity might be linked essentially to modified rather than deficient PPAR*γ* transcriptional regulatory activities as discussed hereafter. Alternatively, altered adipogenesis might be linked to the concomitant perturbed expression of other genes controlling adipocyte differentiation and functions such as genes of the Wnt, Notch, and Sonic Hedgehog signaling pathways [47]. On the contrary to what was observed in obesity, PPAR*γ* expression is decreased in visceral adipose tissues of mouse models of diabetes (db/db), which may directly affect adipocyte differentiation and/or function. The authors showed that this decrease in PPAR*γ* expression is linked to DNA methylation of its promoter [34].Interestingly, recent findings have highlighted a link between epigenetics and metabolism [52] showing that altered metabolism can lead to changes in activity of chromatin-modifying enzymes [53, 54]. Whether and how this could participate in PPAR*γ* abnormal expression in adipocytes remain to be investigated.

### 2.2. Posttranscriptional Regulation of PPAR*γ* Gene Expression in Adipocytes: Role of miRNA

 Epigenomic transitions during adipogenesis often occur at regions distinct from promoters of annotated coding genes [28, 33, 55]. While some of these regions have been defined as enhancers regulating these genes, others are most probably linked to modulation of noncoding RNA (ncRNA) expression. A growing body of evidence points to a major role for ncRNAs in the control of cellular differentiation. Among those are microRNAs (miRNAs), which are short (~22 nucleotides) ncRNAs that posttranscriptionally repress gene expression [56]. By pairing to partially complementary sites in target mRNAs, miRNAs trigger their degradation and/or repress their translation [57]. Several miRNAs play key roles in the control of adipogenesis and adipocyte functions acting as pro- or antiadipogenic factors including miR-30 [58], miR-21 [59], and miR-637 [60] (for review [61, 62]).

Among those, miR-27a/b [63–65] and miR-130a/b [66] are negative regulators of terminal adipocyte differentiation. This inhibition of adipogenesis stems, at least in part, from their ability to prevent the transcriptional induction of PPAR*γ* in preadipocytes. In line, expression of these miRNAs is downregulated during adipogenesis. Both miR-27a/b and miR-130a/b directly target the 3′-untranslated region (3′-UTR) of PPAR*γ* [63, 64, 66]. Additionally, miR-130a/b could also recognize a sequence within the coding region of PPAR*γ* [66].

Interestingly, in agreement with their negative effect on PPAR*γ* expression observed *in vitro*, miR-130a/b expression correlates inversely with PPAR*γ* expression and BMI (body mass index) in abdominal fat depots of female subjects [66]. On the other hand, in contrast to their opposite expression observed during adipogenesis *in vitro*, miR-27a/b and PPAR*γ* are both increased in epididymal fat pads from obese mice (ob/ob) [65]. Therefore, while these studies demonstrate that miR-130a/b play a key role in post-transcriptional regulation of PPAR*γ* expression in adipogenesis and obesity, additional work is required to clarify the role of miR-27a/b in these processes.

 The 5′- and 3′-UTRs of PPAR*γ* mRNA are relatively short (173 and 211 nucleotides long, resp.), which may exclude interaction with a large number of miRNAs [67, 68]. However, since miRNAs can simultaneously target several mRNAs within defined gene networks [69], it would be interesting to analyze whether some additional miRNAs, among those regulating adipogenesis [62], also target PPAR*γ*. Additionally, some miRNAs controlling adipogenesis indirectly regulate PPAR*γ* expression. For instance, miR-31 and miR-155 negatively impact on adipogenesis by directly targeting C/EBP*α* and C/EBP*β* mRNA, respectively, which is secondarily associated with a decrease in PPAR*γ* expression levels [70, 71].

## 3. Chromatin-Based Regulation of PPAR*γ* Activity in Adipocytes

### 3.1. PPAR*γ* Transcriptional Activities Require Chromatin Presetting

Recent insights in our understanding of the transcriptional mechanisms controlling adipogenesis indicate that, reminiscent of other nuclear receptors, PPAR*γ* activities require chromatin presetting. For instance, C/EBP*β* can bind to condensed chromatin in preadipocytes and trigger interdependent recruitment of additional TFs including the glucocorticoid receptor (GR), signal transducers and activators of transcription 5 (STAT5), retinoid-X-receptor (RXR) and C/EBP*δ* to alleviate the repression exerted by SMRT [27, 55, 72]. Altogether, these factors are thought to induce early chromatin opening at enhancers allowing their replacement by PPAR*γ* (and C/EBP*α*) in more mature adipocytes [55, 73, 74]. Consequently, PPAR*γ* binds to enhancers characterized by early nucleosome depletion and presence of histone posttranslational modifications typical of competent/active sites (methylation of histone H3 lysine 4 (H3K4me) and acetylation of histone H3 lysine 9 (H3K9ac)) [28, 33]. These chromatin-based regulatory mechanisms are most probably involved in defining the adipocyte-specific PPAR*γ* transcriptional activities. Indeed, cell type-specific PPAR*γ* binding to chromatin and gene expression regulatory activities depend on different cell-specific collaborating TFs. For example, PPAR*γ* is recruited to enhancers that bind the pioneer factor *PU.*1 in macrophages [75]. 

In addition to enhancers whose chromatin is preset early, PPAR*γ* also binds to many enhancers where chromatin remodelling occurs during adipocyte differentiation [55]. In this case, how PPAR*γ* is directed to these regulatory regions and whether chromatin modifications precede or correlate with its recruitment are not clear yet.

### 3.2. Control of Chromatin Structure by PPAR*γ*

Even though recent studies highlight the need for chromatin presetting in the regulation of PPAR*γ* transcriptional activities, PPAR*γ* activation in turn also leads to additional posttranslational histone modifications. Indeed, PPAR*γ* activation triggers an exchange of interacting cofactors from corepressors to coactivators. These complexes bear enzymatic activities targeting histone acetylation and methylation. For instance, adipogenic differentiation is linked to a shift from complexes containing histone deacetylase (HDAC) to complexes containing hisone acetyltransferase (HAT) activities [76]. This exchange is observed during adipogenesis as well as upon activation of PPAR*γ* with synthetic agonists leading, for example, to increased acetylation of H3K9 at enhancers [75]. It emerges therefore that the activity of PPAR*γ*-dependent enhancers is controlled through sequential stages of chromatin remodelling linked to the hierarchical binding of TFs and cofactors. Chromatin remodelling at these enhancers also involves hydroxymethylation of cytosines through mechanisms that remain to be elucidated [77].

Unlike enhancers whose accessibility is highly variable and cell-type specific, promoters generally lie in open and nucleosome free chromatin regardless of the cell type [78, 79]. However, promoters are controlled by epigenetic modifications that are partly different from those operating at enhancers [79]. For instance, in addition to chromatin remodelling taking place at enhancers, gene activation during adipogenesis also involves modifications at their promoters. This includes methylation of H4K20 by the SET domain containing lysine methyltransferase 8 (Setd8), an enzyme whose expression is induced by PPAR*γ* resulting in the activation of target gene promoters [29]. PPAR*γ*-mediated gene activation during adipogenesis also requires the mediator complex [80]. This complex not only serves as a platform for recruitment of general TFs and RNA polymerase II but can also recruit chromatin remodelers such as chromodomain helicase DNA-binding protein 1 (CHD1) [81]. Altogether, these studies indicate that activation of PPAR*γ* target genes involves a coordinated remodelling of chromatin at both enhancers and promoters. In this context, PPAR*γ*-mediated regulation could involve a defined three-dimensional organisation of chromatin allowing enhancers and promoters to interact, reminiscent of gene activation by other nuclear receptors such as the estrogen receptor *α* [82, 83]. Importantly, PPAR*γ*-bound enhancers could regulate the expression of coding genes important for adipocyte functions both directly and/or indirectly by modulating the levels of miRNAs that control adipogenesis including miR-103 [84, 85]. Obesity leads to altered gene expression profiles in adipose tissue [86, 87]. PPAR*γ* transcriptional activity is exacerbated in obese compared to lean visceral WAT [46]. PPAR*γ* binds to DNA as a heterodimer with RXR, a nuclear receptor subfamily consisting of the isotypes RXR*α*, *β*, and *γ*. Interestingly, RXR*α* protein expression levels are specifically downregulated in visceral white adipose tissue of obese mice and humans through proteasomal degradation. This leads to reduced proportions of the RXR*α*-PPAR*γ* heterodimer and, as a result, to an increased sensitivity to PPAR*γ* agonists, since the SMRT corepressor is more readily dismissed from the remaining RXR*β*-PPAR*γ* complex. Even though not formally demonstrated, the effect of RXR*α* on the interaction with SMRT most probably influences the chromatin structure resulting in a blunted response to PPAR*γ* agonists due to enhanced interaction with HDACs [46]. In addition, the expression of Setd8, which is also increased in white adipose tissue of obese mice [29], may also participate in the strong PPAR*γ* transcriptional response of adipocytes from obese subjects through increased histone methylation. 

## 4. An Integrated View of PPAR*γ* Regulation in Adipocytes

**Figure 2:**
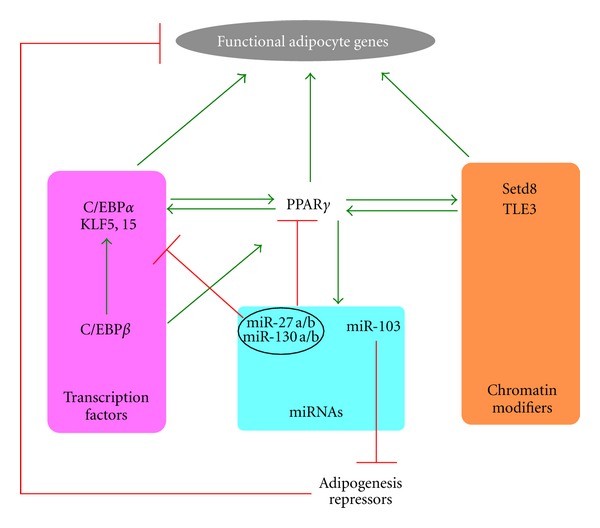
Integrated regulation of PPAR*γ* expression and activity in adipocytes. Schematic showing transcriptional regulators involved in control of PPAR*γ* expression and activity in adipocytes. Green arrows, positive regulation; red bars, negative regulation. See text for further details.

Taken as a whole, regulation of PPAR*γ* expression and activity is a highly integrative process defining an adipogenic transcriptional network involving key cross-regulatory loops between its members (Figure 2). Epigenomic transitions during adipocyte differentiation allow for both temporallycontrolled induction of PPAR*γ* expression and subsequent regulation of its target genes. Chromatin presetting is observed both at *PPAR*γ** and its target genes in preadipocytes and exploited to implement the adipogenic transcriptional program. This process is regulated by an intricate network of hierarchical and combinatorial transcriptional regulatory events. For instance, the transcription factor C/EBP*β* plays a pioneer role early in the course of adipogenesis to induce expression of PPAR*γ*, C/EBP*α*, and KLF5 and 15, which subsequently collaborate to maintain their own expression and activate adipogenic genes. Importantly, this activation requires presetting of chromatin at enhancers by C/EBP*β* [55]. Adipogenesis also involves inactivation of repressors, including miR-27a/b, which target both C/EBP*α* and PPAR*γ* in preadipocytes [63–65]. Among PPAR*γ* target genes are chromatin remodelers including the histone methyltransferase Setd8 [29] and transducin-like enhancer of split 3 (TLE3) [88]. Both factors are involved in a positive autoregulatory loop with PPAR*γ* simultaneously maintaining its high level of expression and induction of target gene expression. While TLE factors are known to have chromatin remodelling activities [89, 90], how TLE3 induces gene expression in adipocytes has not yet been characterized. Overall, PPAR*γ* in adipocytes is therefore controlled by an intricate network of transcriptional regulators that notably license the chromatin structure to allow for appropriate expression and activity of this nuclear receptor.

## 5. Concluding Remarks and Perspectives

We envision that future studies will extend the adipogenic transcriptional network by identifying additional transcriptional regulators and chromatin-associated events controlling PPAR*γ* activities. In addition to a better definition of epigenetic marks involved in this process, studies aimed at identifying mechanisms required for long-range activities of PPAR*γ*-bound enhancers are awaited. This will include a thorough description of the three-dimensional organisation of the chromatin during adipogenesis. Chromosome conformation capture (3C) and its derivatives are recent approaches that have refined our view of the genome spatial organisation and that may prove useful [91]. Taking into account the large diversity of newly discovered ncRNA species, the role of these RNAs in adipogenesis and control of PPAR*γ* is likely at its infancy. Another major question regarding the control of PPAR*γ* activity during adipogenesis relates to the identification of physiologically relevant endogenous ligands [6]. This also requires a better understanding of regulation of PPAR*γ* activities by alternative mechanisms including notably posttranslational modifications [92–95]. Finally, how these regulatory pathways are affected in pathophysiological conditions, such as obesity, will also deserve to be further addressed in order to improve PPAR*γ* targeting strategies. In this context, we will need to better understand the consequences of metabolic perturbations on the enzymatic activities of chromatin modifiers and their consequences for gene transcriptional regulation [52].
